# Health insurance and care-seeking behaviours of female migrants in Accra, Ghana

**DOI:** 10.1093/heapol/czy012

**Published:** 2018-02-15

**Authors:** Samantha R Lattof

**Affiliations:** Department of Social Policy, London School of Economics and Political Science, Houghton Street, London WC2A 2AE, UK

**Keywords:** Health insurance, health care-seeking behaviour, determinants, perceptions, poverty, access, urban health, gender, informal sector, population movement

## Abstract

People working in Ghana’s informal sector have low rates of enrolment in the publicly funded National Health Insurance Scheme. Informal sector workers, including migrant girls and women from northern Ghana working as head porters (*kayayei*), report challenges obtaining insurance and seeking formal health care. This article analyses how health insurance status affects *kayayei* migrants’ care-seeking behaviours. This mixed-methods study involved surveying 625 migrants using respondent-driven sampling and conducting in-depth interviews with a sub-sample of 48 migrants. Analyses explore health status and health seeking behaviours for recent illness/injury. Binary logistic regression modelled the effects of selected independent variables on whether or not a recently ill/injured participant (*n* = 239) sought health care. Although recently ill/injured participants (38.4%) desired health care, less than half (43.5%) sought care. Financial barriers overwhelmingly limit *kayayei* migrants from seeking health care, preventing them from registering with the National Health Insurance Scheme, renewing their expired health insurance policies, or taking time away from work. Both insured and uninsured migrants did not seek formal health services due to the unpredictable nature of out-of-pocket expenses. Catastrophic and impoverishing medical expenses also drove participants’ migration in search of work to repay loans and hospital bills. Health insurance can help minimize these expenditures, but only 17.4% of currently insured participants (58.2%) reported holding a valid health insurance card in Accra. The others lost their cards or forgot them when migrating. Access to formal health care in Accra remains largely inaccessible to *kayayei* migrants who suffer from greater illness/injury than the general female population in Accra and who are hindered in their ability to receive insurance exemptions. With internal migration on the rise in many settings, health systems must recognize the varied needs of populations in multi-ethnic and multilingual countries to ensure that internal migrants can access affordable, quality health services across domestic borders.

Key Messages

- Less than half of recently ill or injured migrants sought health care, citing time away from work and lack of a translator as problems.
- Odds of seeking health care are directly related to migrants’ informal sector earnings on a bad market day.
- Although 58.2% of migrant respondents reported being currently insured, only 17.4% reported holding a valid health insurance card.
- It is paradoxical that migrants from northern Ghana, where the concentration of health facilities is lowest, cannot and do not access formal health services in Accra, where Ghana’s concentration of health facilities is highest.

## Introduction

As international and internal migration grow in scope and complexity, migrants are at risk of exclusion from universal health coverage (UHC). In recognition of the links between health and development, the Sustainable Development Goals recognize UHC as underpinning and balancing all health targets (WHO, 2015). UHC ensures that everyone can access affordable, quality, essential health services and requires regular monitoring of progress in order to adapt UHC to local demographic, epidemiological and technological conditions (WHO and World Bank, 2015).

The inclusion of international migrants in regional health insurance schemes, such as the European Health Insurance Card, moves some UHC systems closer towards providing truly universal care; however, many world regions lack such schemes, leaving international migrants poorly served by national health strategies or excluded all together. Internal migrants may also become excluded from UHC or discriminated against when seeking health care. Pricing health insurance and health care expenses based on geography rather than a sliding socioeconomic scale may price rural-to-urban migrants out of health services.

In Africa, most national health strategies integrate UHC as a goal, yet progress towards achieving UHC has been uneven. Critical gaps remain in health service coverage, and out-of-pocket health payments push 11 million Africans into poverty annually (World Bank, 2016). Among African countries leading UHC implementation, Ghana is noted for its efforts to reduce financial barriers for the poor (World Bank, 2016). Before establishing the National Health Insurance Scheme (NHIS) in 2003, Ghana’s health system was based on user-fees that were often a barrier to care for the poor. With the NHIS, basic health care became available to all citizens after a processing fee and an initial premium determined by each district’s health insurance scheme. Value-added tax funds most (∼70%) of the centrally funded scheme with additional contributions from the formal sector Social Security and National Insurance Trust (SSNIT) (20–25%), non-SSNIT contributor out-of-pocket premiums (5%) and other sources (∼2–5%) (Odame *et al.*, 2014). Access to health care has improved; however, Ghana’s efforts to achieve UHC remain challenged by financing and equity (Garshong and Akazili, 2015). Health service use and access to care remain inequitable with poor people burdened disproportionately by the processing fees and premiums.

NHIS membership has been found to be positively associated with urban residence, higher educational attainment and higher socioeconomic status (Akazili *et al.*, 2014). Three in five Ghanaians reported that they are unable to afford the insurance renewal payment, and 71.6% of the uninsured report that they cannot afford the premium (Jehu-Appiah *et al.*, 2012). Although several categories of people may receive an exemption from the premiums (e.g. children, elderly, indigents and pregnant women), many Ghanaians have not utilized these exemptions (Kanchebe Derbile and van der Geest, 2013). Individuals receiving premium exemptions must pay the processing or renewal fee unless exempt due to pregnancy, mental disorder or poverty. NHIS enrolment of people working in Ghana's informal sector remains low. Without clear guidelines/criteria for identifying ‘indigents’ and without detailed costing analysis data, few benefit from the indigent exemption (Jehu-Appiah *et al.*, 2010, Kotoh and van der Geest, 2016).

Annual NHIS premiums range from GHȻ 7.20–48.00 (US$1 = GHȻ 4.14 as of 1 January 2017) depending on individual socioeconomic status, though most districts set a flat rate due to challenges in assessing socioeconomic status. This flat rate premium and variable processing fee (GHȻ 4 or more) may disproportionately burden members of Ghana’s informal sector. The informal sector employs two-fifths of employed Ghanaians aged 15 years and older; sex-disaggregated data reveal that the informal sector employs a larger percentage of currently employed females (47.8%) than males (35.5%) (Ghana Statistical Service, 2014b).

In low- and middle-income countries (LMICs), relatively little is known about disadvantages arising from informal sector employment, migrant status and gender. Migrant girls and women from northern Ghana who work in the capital as head porters (*kayayei* (plural), *kayayoo* (singular)) report challenges obtaining insurance and accessing health care (Sabutey, 2014) as do other members of Ghana’s informal sector (Alfers, 2013). Although impoverished, young and pregnant migrant populations may qualify for NHIS exemptions, they still struggle to access formal health services (Yiran *et al.*, 2015). These struggles occur within the context of increasing urbanization. Ghana’s capital, Accra, is one of the fastest growing cities in West Africa (African Development Bank Group, 2014). Two in five current residents in the Greater Accra Region migrated from one of Ghana’s other nine regions, and inter-regional migration is expected to continue as Ghana’s economy grows (Ghana Statistical Service, June 2013). Such difficulties accessing health services warrant greater attention to the interplay between migration, informal sector employment and gender.

The aim of this article is to determine the ways in which health insurance status affects female migrants’ care-seeking behaviours, using the migrant *kayayei* in Accra as a case study. Why do female migrants working in the informal sector (not) use health services in Accra? What role does health insurance play in female migrants’ decision and ability to access care?

## Methods

### Study design and setting

This study examined contemporary north–south migration in Ghana at the national level and among a population of female migrants working as *kayayei* in Accra. The primary data were gathered using a fully mixed concurrent equal status design (Leech and Onwuegbuzie, 2007) to collect quantitative and qualitative data in Agbogbloshie/Old Fadama, Accra between March and April 2015. The Agbogbloshie/Old Fadama area is Accra’s largest informal settlement and home to ∼80 000 residents from inside and outside of Ghana; 72.4% of residents migrated from Ghana’s three northern regions (Housing the Masses, 2010). The research involved collecting primary survey data on 625 migrant *kayayei* living in Accra using respondent-driven sampling (RDS).

Seven data collectors who self-identified as *kayayei* were hired and trained to administer questionnaires that included questions on women’s health, families, employment and migration. After each questionnaire was completed, the author immediately reviewed the survey for quality and to purposively select participants (*n* = 48) for an in-depth interview (IDI) based on responses to a question about illness/injury within the 2 weeks preceding the survey (hereafter ‘recent illness/injury’).

The author conducted IDIs, with translation facilitated by one of the data collectors, among 48 *kayayei* who experienced a recent illness/injury. These interviews explore migration histories, experiences of recent illness/injury and use of health services. Quantitative and qualitative data are integrated thematically in the analyses. To address validity and reliability of these results as well as to mitigate the biases arising from single-method research, the author triangulated the results across methods to indicate where findings converge or diverge (Jick, 1979).

The London School of Economics Research Ethics Committee (18 November 2014) and the University of Ghana’s Noguchi Memorial Institute Institutional Review Board (Protocol Number 065/14-15) reviewed and approved this research. Approval was also received from local community members, including the Board of Directors of the Kayayei Youth Association (KYA), *kayayei* leaders, the Director of Social Welfare and the chief of Yam Market. No participant could participate without informed consent.

### Methodology for survey

The survey questionnaire included some questions from the Ghana Living Standards Survey 6 (GLSS6) and the Ghana Demographic and Health Survey to allow for selected item comparability. Researchers at the LSE and the University of Ghana, the KYA, data collectors and *kayayei* leaders provided feedback on a draft of the questionnaire. The research team pre-tested and piloted the questionnaire in the field in March 2015 to verify the questionnaire structure, formulation of questions, appropriate language and terminology, completion times, range of variation in response variables and respondent understanding. The pilot study revealed that the term ‘sister’ was causing confusion among data collectors, since in this setting ‘sister’ is often used to refer to a close female friend. All survey references to ‘sister’ were changed to ‘biological sister’ to prevent confusion. The resulting survey tool consisted of a 25-page printed questionnaire with 10 modules asking a total of 236 possible questions. It generated primarily quantitative data, with a limited number of open-ended questions.

As a ‘hard-to-reach’ population whose members are stigmatized and for whom a sampling frame is not available, *kayayei* were recruited through their social networks using RDS. RDS, a variation on chain-referral sampling methods like snowball sampling, was originally developed in the USA as a method to sample ‘hidden’ populations such as people living with HIV/AIDS and injecting drug users (Heckathorn, 1997). Unlike chain-referral methods that lead to statistical difficulties making inferences from the sample, RDS includes a mathematical model to account for the non-random way in which the sample was collected (Heckathorn, 1997). It leads to a weighted sample that has been proven to be unbiased for samples of meaningful size regardless of how the researcher selects the initial ‘seeds’ who initiate the study (Salganik and Heckathorn, 2004).

Researchers have revised the RDS methodology over time to adjust components such as the calculation of standard errors, new estimators, a bootstrap method for constructing confidence intervals and larger design effects (Heckathorn, 2002, 2007; Salganik, 2006; Volz and Heckathorn, 2008; Wejnert *et al.*, 2012). Even with these revisions, the RDS methodology validity warrants caution and is not appropriate for all studies. Simulations find that the RDS assumptions, discussed in the limitations section below, may be unrealistic and can lead to biased estimates (Gile and Handcock, 2010). Other simulations critique RDS’s ‘misleadingly narrow’ confidence intervals that public health researchers might find unsuitable for disease surveillance (Goel and Salganik, 2010).

Formative research conducted in advance of the survey generated evidence on crucial RDS implementation factors including: appropriate incentives; participants’ personal network sizes; hours and days during which to conduct the study; coupon design; the survey instrument; and participants’ prior experiences with researchers, outsiders and census enumerators. This formative research also confirmed that Ghana’s migrant *kayayei* meet the criteria to sample using RDS: they can be clearly defined; they recognize one another as part of the group; and, they have the characteristics of a social group (i.e. *kayayei* both identify and interact with each other) (Friberg and Horst, 2014). In addition, key informants revealed that *kayayei* migrants had sufficiently dense network ties to facilitate chain referral.

Based on this formative research and a review of studies with comparable estimated sample sizes, the author identified ten seeds with support from the KYA (Kubal *et al.*, 2014). Seeds were selected to ensure diversity across characteristics such as the current length of time spent in Accra, the number of migrations made to Accra, primary worksite in Accra and homelessness. Prior studies indicate that most North–South female *kayayei* migrants are from the Northern Region and Muslim, so a higher proportion of seeds reflected these characteristics. Four seeds failed to generate sufficient recruitment chains. Since the remaining six seeds were growing sufficiently, only two replacement seeds were added to the study.

Recruits were invited to participate in the study if they:

- Were a girl or woman;
- Currently worked as a *kayayoo* in Accra;
- Migrated to Accra from one of the three northern regions (Upper East Region, Upper West Region, or Northern Region);
- Held a valid study participation coupon as required by RDS.

Recruits were ineligible to participate if they:

- Were not from the Upper East Region, Upper West, or Northern Regions;
- Did not work as a *kayayoo*;
- Did not have a coupon or held an expired coupon;
- Had already participated.

Estimating sample size for RDS cannot be directly calculated a priori, as sample size depends on the collected network data that are then used to calculate sampling weights (Heckathorn, 2002). However, researchers may generate sampling estimates to assist in planning and implementing their studies by calculating the sample size for simple random sampling and then adjusting the calculation for the design effect of RDS (Wejnert *et al.*, 2012). To achieve the same power as a simple random sample, the RDS literature recommends selecting a design effect (deff) between two and ten that measures increased variation of the estimates (Salganik, 2006; Goel and Salganik, 2010; Wejnert *et al.*, 2012). This study used a more conservative design effect of 10 and a SE of 0.05. Since one of this study’s goals was to examine the prevalence of illness or injury in the last 2 weeks among *kayayei* migrants in Accra, the author calculated RDS sample size (*n*) for cases involving estimation of prevalence (Salganik, 2006):

$$n=deff\cdot \frac{P_{A}(1-P_{A})}{(se(\hat{P_{A}}){)}^{2}}$$

This study selected a prevalence of interest (*P*_A_) based on data from the fifth Ghana Living Standard Survey that reported a 16.0% prevalence of illness or injury in the last 2 weeks among females aged 20–49 years in Accra (Ghana Statistical Service, 2008). The author planned for an estimated sample of 538 *kayayei* that was thus ten times as large as a simple random sample in order to achieve the same statistical power.

### Analyses for survey

RDS analyses were conducted using the Respondent Driven Sampling Analysis Tool (RDSAT) Version 7.1 (Volz *et al.*, 2012). Partition analyses for categorical and continuous variables in RDSAT used the recommended options for optimal accuracy: ‘dual component’ estimate for average network size with a mean cell size of 12, enhanced data-smoothing algorithm type, 16 000 re-samples for Bootstrap, and alpha = 0.025 (Spiller *et al.*, 2012, Heckathorn, 2007). The analyses are based on six assumptions about RDS: (1) respondents have reciprocal relationships with one another; (2) respondents’ social networks are dense enough to sustain a chain-referral process; (3) each respondent recruits a single peer; (4) respondents recruit randomly from their networks; (5) respondents can accurately report their personal network size to data collectors; and (6) sampling occurs with replacement (Salganik and Heckathorn, 2004; Heckathorn, 2007). Formative research with *kayayei* produced confidence in the first three assumptions. Factors such as incentives, study location(s) and research topic may influence to whom participants give their coupons, thus affecting the fourth assumption (Heckathorn, 2007). The author discussed these factors with key informants during the formative research period in an attempt to minimize potential violation of the fourth assumption. Data collectors also encouraged participants to recruit randomly when giving oral recruitment instructions. The research team tested the fifth assumption extensively during data collector training. Most RDS studies violate the sixth assumption, since participants cannot participate again by design. Biases from violating this particular assumption remain poorly understood (Goel and Salganik, 2009; Volz and Heckathorn, 2008), though this assumption is relaxed among samples that are relatively small compared with the total population’s size (Volz and Heckathorn, 2008).

All surveys were manually entered into SPSS 22.0 software for logistic regression analyses. Binary logistic regression modelled the effects of selected independent variables on whether or not a recently ill/injured participant (*n* = 239) sought health care. Selection of the independent variables was based on a literature review of barriers and determinants of care as well as participant-reported factors from the survey and IDIs.

### Methodology for IDIs

The author and translator conducted interviews inside the KYA office on Sundays and at times when the office was closed for business in order to provide a quiet, private setting. The IDIs involved a semi-structured interview guide on: demographics, migration history, experience (not) seeking care for recent illness/injury, health knowledge, use of health services, fertility and ties to family/friends in the North. Interviews averaged 59 min and were linked to the participants’ surveys using each participant’s unique RDS identification number. Six exploratory pilot interviews explored appropriate language and terminology, assessed validity of the survey questions, tested the flow of the questions, and formulated the final interview guide. As these pilot interviews did not result in significant changes to the interview guide or interview method, they are included in the final sample.

The IDI sample (*n* = 48) was selected from survey participants using a non-probabilistic stratified purposive sampling approach. This approach allows for comparisons between subgroups while displaying variation on migration and reproductive health (Patton, 2002). Not only must the sample have ‘symbolic representation’, but it must also illustrate the diversity within the population’s boundaries (Ritchie *et al.*, 2003). Based on these requirements and characteristics suggested by the literature, the author selected survey participants based on whether or not the participant sought medical care for her recent illness/injury, number of migrations to Accra and age group (younger half of the sample or older half of the sample). Access to participants’ demographic information in their surveys permitted refined sampling.

All interviews were audio recorded and, when not in English, translated into English during the interview. English content was then transcribed verbatim in transcripts that were entered into NVivo 11 software for thematic coding and analysis. Where translation was required, the final transcripts reflect the best English that the translator knows and are shaped by her work and experience as a *kayayoo*, albeit one of the few with a high school education. To ensure translation quality, one in every four interviews was selected for verification. A second multi-lingual data collector listened to the 12 interview recordings and documented the instances in which she disagreed with the translation. The second data collector approved of the translation quality, noting several minor differences that she believed did not warrant re-translation. For three interviews, she offered additional insight beyond what the participant and translator provided (e.g. determining the name of a hospital based on the location’s description).

Influenced by grounded theory, initial analyses were guided by a codebook based on themes emerging from the pilot study and subsequent interviews. Analyses presented here focus on the ways in which health insurance and other factors affect female migrants’ care-seeking behaviours. The anonymized, illustrative quotations in this article come from a range of interviews to explore heterogeneity.

## Results

### Sample characteristics

The migrant *kayayei* in this study had a mean age of 25.2 years (SD 8.1 years); four out of five migrants were aged 15–34 years (Table 1). Most practiced Islam (88.2%) and were married or living with a partner (60.2%). Approximately two-thirds of survey participants identified as Mole-Dagbani from the Northern Region, followed by 13.3% as Sissala from the Upper West Region and 9.1% as Frafra from the Upper East Region. One-third of survey participants were in Accra on their first migration. One-third of participants had returned to Accra on a second migration, and one-third had migrated to Accra three or more times. In stark contrast to girls and women in Accra (0.4%), 73.3% of *kayayei* migrants had not completed any formal schooling.

**Table 1:** Demographic characteristics of recently ill/injured participants compared with the entire study sample and to GLSS6 data on females residing in Accra^a^

Characteristics	Recently ill/injured participants (*n* = 239) (%)	Entire sample (*n* = 625) (%)	GLSS6 data (*n* = 3466) (%)
Highest educational attainment^b^			
Kindergarten			31.9
Primary	20.9	17.3	14.7
Middle/Junior Secondary School	8.8	7.4	28.2
Secondary/Senior Secondary School	2.9	2.1	13.2
Vocational/Technical/ Commercial			4.1
Teacher Training/Nursing			1.5
Post-Secondary Diploma			2.7
Bachelor Degree			2.8
Post-Graduate Degree			0.5
None	67.4	73.3	0.4
Age group (in years)			
0–4			10.8
5–9			9.9
10–14	4.6	6.9	11.3
15–19	13.0	15.0	10.2
20–24	25.9	25.9	9.3
25–29	19.2	19.5	10.2
30–34	19.2	18.9	8.3
35–39	8.8	5.6	7.1
40–44	7.9	6.2	6.3
45–49	0.8	1.1	4.7
50+	0.4	0.8	11.9
Homeless			
Yes	10.5	17.6	
No	89.5	82.4	
Number of migrations to Accra			
1	31.4	33.4	
2	35.1	35.4	
3	18.0	17.1	
4	6.7	7.0	
5	3.8	3.4	
6	3.8	2.9	
7	1.3	0.8	
Ethnic group			
Mamprusi	8.4	5.9	0.4
Sissala	15.5	13.3	0.3
Ashanti (Asante)		0.2	7.8
Guan	0.4	0.2	0.3
Mole-Dagbani, Dagomba	51.9	67.2	1.7
Grussi	4.6	1.8	0.3
Gruma	1.3	0.5	0.1
Konkomba	0.8	0.5	0.0
Dagaba^c^	0.4	0.2	0.7
Kusasi	0.8	0.6	0.6
Mandé	0.4	0.2	–
Frafra	15.1	9.1	1.2
Walla^c^	0.4	0.2	
Gonja		0.2	0.6
Hausa-Dagomba		0.2	
All other tribes originating in Ghana			83.9
Tribes originating outside Ghana		–	2.1
Religion			
Catholic	15.5	8.6	5.7
Anglican	1.3	0.5	–
Methodist	0.8	0.3	–
Presbyterian	2.5	1.4	–
Pentecostal/Charismatic	1.3	0.8	51.9
Muslim	78.2	88.2	11.0
Traditional/Spiritualist	0.4	0.2	0.8
Protestant			19.0
Other Christian			10.1
None			1.4
Marital status			
Never married	25.9	28.6	40.9
Engaged to be married	3.8	3.8	
Married or living with partner	60.3	60.2	43.3
Separated	2.1	1.8	3.3
Divorced	1.3	0.6	4.8
Widowed	6.7	5.0	7.6

a Comparison data in this table come from a 10% microdata sample of the GLSS6 and are limited to girls and women residing in the Greater Accra Region.

b GLSS6 data on highest educational attainment are for household members aged 3 years or older.

c GLSS6 data combine the ethnicities Dagaba, Walla and Lobi into one group. These joint data are reported once in this table under Dagaba.

Participants cited numerous triggers to migration, including the need to pay for health care. Family medical expenses drove several participants’ migrations in search of work to repay loans and hospital bills:

*So when the car knocked my husband [in a hit-and-run accident], I was taking care of my husband. I sold all my property. My cloth, my bowls. I sold it all to take care of him at the hospital. So when he was healthier, he said that I should come to Accra so that maybe I can get something good to bring back. It’s better than all of us sitting in the house* (Mole-Dagbani woman aged 30, mother of six children).

*What brought me to Accra is because of money, and the serious reason is one of my brothers was sick with his leg. They wanted to cut the leg, so they sent him around to hospital. When they sent him, he didn’t get good treatment, so they went to some place again for hospital and they said they can treat him but the bill is 500 cedis. That’s an issue. […] My family, all of them they are poor. So I said I would come [to Accra for work] because I’m younger than all of them. I will come and work so that they can treat my junior brother* (Mole-Dagbani woman aged 25, one of five siblings).

Four in five survey respondents assessed their pre-migration health status, referring to their health status prior to the most recent migration, as good or very good (Table 2). Upon moving to Accra, self-reported health status declined for many *kayayei*. One in three participants considered their post-migration health status as bad or very bad, and 38.4% of participants reported a recent illness/injury.

**Table 2:** RDS sample proportions and population proportion estimates for key health variables

	Sample population proportions	Estimated population proportions (95% CI)
Illness or injury in last 2 weeks	
Illness	35.2	21.5 (17.8, 25.8)
Injury	2.6	1.6 (0.6, 3.1)
Both	0.6	0.1 (0.0, 0.3)
Neither (healthy)	61.6	76.8 (72.2, 80.7)
Current self-reported health status (Accra)	
Very good	9.6	9.6 (6.4, 13.5)
Good	35.4	46.2 (40.7, 52.5)
OK	19.0	20.5 (15.7, 25.1)
Bad	20.3	12.7 (9.9, 15.9)
Very bad	15.7	10.9 (8.0, 14.0)
Pre-migration self-reported health status (North)	
Very good	38.9	35.0 (30.1, 40.0)
Good	43.2	42.8 (37.4, 48.5)
OK	5.3	6.2 (3.2, 9.9)
Bad	9.3	13.3 (9.2, 17.5)
Very bad	3.4	2.8 (1.4, 4.3)

Population proportions are reported per 100 individuals.

### Care-seeking for recent illness/injury

Among survey respondents who reported experiencing a recent illness/injury, less than half sought care. Seeking care included consulting with a formal health practitioner (e.g. doctor, nurse and pharmacist) or an informal provider (e.g. peddlers in the market). Qualitative and quantitative evidence show that lack of money was the main barrier to seeking care. In the survey, 95.8% of recently ill/injured participants reported lack of money as a ‘big problem’ to seeking care.

Interviewer: *If the neck pain is bad enough that it stops you from working, why did you not get care?*

Respondent: *I want to. I want to get money enough so that I can go and do the national health insurance so that I will go to hospital* (Sissala woman aged 19).

Interviewer: *Why did you not go get medical care for the cut on your foot?*

Respondent: *I don’t have money to go.*

Interviewer: *Could you have borrowed the money?*

Respondent: *If I want to go, they will not give me [money]. Nobody will give me.*

Interviewer: *Do you have health insurance?*

Respondent: *Yes.*

Interviewer: *Could you have used your health insurance to go to hospital?*

Respondent: *I didn’t bring it here.*

Interviewer: *Why did you not bring it?*

Respondent: *I forgot it* (Mole-Dagbani girl aged 14).

To live and work in Accra, participants paid daily living expenses that included a market tax to the Accra Metropolitan Assembly ticket collectors (GHȻ 0.50), water for drinking and bathing (median GHȻ 1.00) and use of the toilet (median GHȻ 0.50). Four in five participants rented shelter, paying a daily median of GHȻ 0.43 for space in a shared room. Any remaining income beyond these expenses was used for food, medical expenses and remittances. Having no money was the largest barrier to seeking care (97.2%) among participants who did not consult anyone for their conditions. Over half of recently ill/injured participants did not seek a consultation (Table 3). Among ill/injured *kayayei* migrants who sought care, the majority consulted drug/chemical sellers (53.3%) or doctors (31.4%).

**Table 3:** RDS sample proportions and population proportion estimates for key health consultation variables

	Sample population proportions	Estimated population proportions (95% CI)
Consult for recent illness		
Yes	43.5	39.2 (29.5, 54.8)
No	56.5	60.8 (45.2, 70.5)
Who consulted first		
Doctor	31.4	19.8 (6.9, 33.4)
Nurse	8.6	17.1 (1.7, 38.3)
Pharmacist	6.7	10.5 (0.0, 16.5)
Drug/chemical seller	53.3	53.3 (39.7, 72.4)
Why no consult		
No money	97.2	97.8 (92.8, 1.0)
Health insurance expired	2.8	2.2 (0.0, 7.2)

Population proportions are reported per 100 individuals.

Seeking medical care for a recent illness/injury (Table 4) was significantly associated with the amount of money an ill/injured participant-reported earning on a bad day, such as when the market was slow. On bad market days, participants reported earning an average of GHȻ 5.35 with 6.1% of participants reporting no income. In contrast, participants reported earning an average of GHȻ 12.52 on good market days. Good market days, however, appeared far less frequently than bad market days, according to participants. With each cedi that an ill/injured participant earned on a bad day, her odds of seeking health care increased 1.301 times (*B* = 0.263; *P* = 0.005). Ill/injured participants were substantially less likely to seek health care if they considered taking time away from work (*B* = −1.490; *P* = 0.028) and lack of a translator as problems (*B* = −3.179 *P* = 0.001). Arm or leg pain increased participants’ odds of seeking medical care by 7.119 times (*B* = 1.963; *P* = 0.017) whereas participants experiencing fever were significantly less likely to seek medical care (*B* = −3.786; *P* = 0.041).

**Table 4:** Summary of logistic regression analysis for variables predicting whether a recently ill or injured survey participant (*n* = 239) sought medical care

Independent variables		*B*	SE *B*	*P*-value	Exp(*B*)
Age (in years)		0.004	0.036	0.919	1.004
Highest level of school completed	Never attended school			0.810	
	Primary	0.326	0.630	0.605	1.386
	Middle/JSS^a^	–0.460	0.912	0.614	0.632
	Secondary/SSS^b^	–1.063	1.647	0.518	0.345
Type of illness or injury	Neck and/or back pain			0.100	
	Stomach pain	–0.102	0.957	0.915	0.903
	Arm or leg pain	1.963	0.823	0.017	7.119
	Full body pain	20.416	15 603.022	0.999	735 545 361
	Fever	–3.786	1.850	0.041	0.023
	Headache	1.333	0.739	0.071	3.793
	Chest pain	0.699	0.722	0.333	2.012
	Eye problem	20.113	27 862.283	0.999	543 260 199
	Starvation and/or dehydration	–0.309	2.861	0.914	0.734
Money earned in Accra on a bad day		0.263	0.094	0.005	1.301
Taking time away from work to seek care		–1.490	0.676	0.028	0.225
No translator at facility		–3.179	0.928	0.001	0.042
Do you hold a valid health insurance card?	Yes			0.011	
	Yes, card in Accra not seen/lost	–0.339	0.883	0.701	0.713
	No, expired	–0.354	0.787	0.653	0.702
	Yes, card left in North	1.139	0.672	0.090	3.124
	No, never registered	–2.195	1.280	0.086	0.111
Current self-reported health status in Accra	Very good			0.239	
	Good	0.109	1.354	0.936	1.115
	OK	1.576	1.360	0.246	4.835
	Bad	0.100	1.315	0.939	1.105
	Very bad	–0.543	1.427	0.704	0.581
Pre-migration self-reported health status in the north	Very good			0.097	
	Good	1.651	0.682	0.016	5.210
	OK	0.862	1.475	0.559	2.367
	Bad	–1.103	1.186	0.352	0.332
	Very bad	–18.522	40 192.970	1.000	0.000
Constant		0.888			
Cox and Snell *R*^2^		0.545			
Nagelkerke *R*^2^		0.732			

This model accurately predicts 87.8% of cases.

a Junior Secondary School.

b Senior Secondary School.

Prior experiences with the formal health system, including stigma and discrimination, may lead participants to seek informal care outside of health facilities. Providers perceived *kayayei* migrants as being unable to afford services, which migrants perceived as affecting their quality of care:

*When I went to the doctor, they were complaining*, *‘that’s why you kayayoo people, that’s why you people if you’re sick and you don’t have money, you’ll be coming here and disturbing people’* (Frafra woman aged 21).

*In the north, because they know that that is my town, I’m staying there, they know that oh, I am also part of the community in the area. So if you go to the hospital because even north, if you have the national health insurance, they look after you free. But because of here, even if you go there, they recognize that we are kayayoo people. So they are not serious to take care of us. They will just, because they know that ‘oh, if I just call you come inside [for treatment], you don’t have money’* (Mole-Dagbani woman aged 30).

As a result of large out-of-pocket expenses not covered by national health insurance or prescriptions requiring purchase outside the health facility, ill/injured participants often sought less expensive informal care or declined care:

*The health insurance help is the most important for the operation side and water* [fluid therapy] *side. But for them to give you a good medicine, they will not give you. They will rather write it for you to go and buy* (Frafra woman aged 25).

Interviewer: *Why did your health insurance not cover it [Hepatitis B]?*

Respondent: *I used it. I used the health insurance.*

Interviewer: *They still took money?*

Respondent: *Yes. That is why if I get sick, I will go to the drugstore. Because if you use the health insurance, they will still collect some money from you.*

Interviewer: *Even with health insurance, they were going to take 130 cedis?*

Respondent: *Yes. For the [Hepatitis B] labs. Sometimes they don’t want to give you this thing. They don’t want to tell you to bring the money. They will write the medicine they are supposed to give you and they give it to you to go and buy. If you don’t have money, how can you do that?* (Mole-Dagbani woman aged 32, diagnosed with Hepatitis B during antenatal care).

The interviewee above with Hepatitis B declined to seek additional treatment for her illness due to the out-of-pocket expenses she would incur. She went on to deliver twins, one of whom died in the days following childbirth.

With hospitals perceived as the most expensive places to seek care, even with health insurance, ill/injured participants sought less expensive forms of care from pharmacies and roaming petty traders. Migrants used local community pharmacies and drugstores that were easily accessible on foot and where vendors might speak northern dialects. Petty-traders provided a source of even less expensive care, though the quality of medicines they offered was unknown. Migrants weighed this risk against a lack of money and relied upon prayers, personal experiences and friends’ recommendations to select medications and vendors.

*Because I know that the hospital money is very costly, that is why sometimes I buy medicine from those who are roaming. So that if I just take some medicine and it cool me, I will work again. When it [the pain] starts again, I will go and buy the medicine. But it’s not nicer than the hospital because they [roaming vendors] cannot check you. But because I don’t have money, that is always why I buy the small small drugs outside* (Mole-Dagbani woman aged 41).

Respondent: *The people who are at the drugstore, sometime if you go, the medicine price is high. And those who are going round, sometimes their medicine price is low. So that is why sometimes you buy from them.*

Interviewer: *I’ve heard that sometimes the medicine they sell is expired. It’s old, or it is fake. Have you heard that?*

Respondent: *We hear that. We hear all this but because our money is not enough, we are always praying to god that if we take this medicine, we will be healthy* (Grussi woman aged 22).

### Health insurance status

Most participants had been insured through Ghana’s NHIS at least once in their lives (Table 5). Although health insurance can help minimize health expenditures, only 58.2% of all participants (*n* = 625) were currently insured. Of those currently insured, 17.4% reported holding a valid health insurance card. Women reported that health insurance cards were occasionally burned in room fires or stolen along with the contents of women’s purses. Participants also reported losing their cards or forgetting them when migrating, restricting access to formal health care in Accra.

**Table 5:** RDS sample proportions and population proportion estimates for key health insurance variables

	Sample population proportions	Estimated population proportions (95% CI)
Ever insured		
Yes	77.4	76.5 (71.4, 81.2)
No	22.6	23.5 (18.8, 28.6)
Currently insured		
Yes	58.2	52.7 (46.3, 61.2)
No	41.8	47.3 (38.8, 53.7)
Hold a valid insurance card		
Yes	17.4	10.8 (6.6, 15.6)
No (card is lost, expired, or left in north)	82.6	89.2 (84.4, 93.4)

Sample population proportions are reported per 100 individuals.

Leaving a health insurance card in the north was common, particularly if the card was expired. Long processing delays to renew an expired card also meant that participants migrated south without their health insurance cards, as the need to start earning money outweighed waiting for their cards. Others forgot their cards accidentally when packing. One participant who migrated south to leave an abusive relationship reported that she left her health insurance card at her husband’s house because of the fighting. There was also uncertainty about whether health insurance and insurance cards from the north would work in the south:

*Because I did it at the north, I was thinking if I bring it here, will it work here, or it will it not work? I left it in the north. I said maybe in the north is different and Accra is different* (Mole-Dagbani woman aged 33).

Nearly all participants without health insurance recognized the value of having health insurance. A lack of money, however, prevented them from registering in the north or in Accra. Some participants were unaware of the costs and believed that they needed to save GHȻ 80–100 before they could register with the NHIS in Accra; these figures greatly exceed the GHȻ 25–30 that other *kayayei* report spending on health insurance in Accra. This perception of health insurance costing more in Accra extended to maternity care services that are supposed to be exempt from fees:

*Because north, they give everything free to us. Card, antenatal care, and what they give is free. But here, they don’t give us free* (Mole-Dagbani woman aged 24).

Some participants perceived that paying for medical expenses out of pocket would result in better quality and more timely care than using national health insurance:

*If you go without the health insurance, they will treat you well. So you get better care if you have no health insurance. Because they know you can pay* (Frafra woman aged 25).

*Even the national health insurance, they know they will not collect plenty money. They will let you sit down* [wait] *and they will take those who don’t have national health insurance* (Mole-Dagbani woman aged 41).

Most participants without insurance or with expired insurance lacked the money to register or renew their policies. One participant reported that an NGO in Accra came to enrol *kayayei* in the NHIS for free, taking their photographs and completing the forms. The organization never returned with health insurance cards. A similar situation was reported in the north, where a pregnant woman was told NHIS employees would visit her village hospital to register local residents; she reported that the registration never happened.

Among participants who had ever given birth (70.6%), more than four-fifths (88.9%) registered for health insurance the last time they were pregnant. Women who did not register when pregnant most often reported that they were unable to afford insurance (4.6%), did not think it was for them (3.0%), or did not know about it (1.9%). Under the NHIS guidelines that include free antenatal and childbirth care, pregnant women are exempt from both the health insurance premium and registration fee. Some mothers reported, however, that they had to pay for health insurance when pregnant or that the cost of health insurance when pregnant led them not to seek insurance. However, the majority of participants reported no problems registering under the pregnancy exemption or accessing free maternity services, apart from the fact that the free policy ends shortly after childbirth:

*When I was pregnant and they give me the national health insurance, everything they were doing for me free. Medicine and these things. When I was going to give birth, free. When I give birth, it finished. That is all the national health insurance. I have to go and do another one* (Mole-Dagbani woman aged 26).

Health insurance exemption for pregnant women ends when the infant is 3 months old; mothers must purchase new policies for themselves and also pay the card processing and annual renewal fees for their babies who are exempt from premiums until age 18 years. Over half of mothers (54.3%) report being currently insured; however, only 13.6% of these currently insured mothers report holding valid insurance cards needed to access care. Although some mothers can afford to pay health insurance expenses for themselves and their children, many poor mothers with limited resources find themselves in a position where they have to choose whom to cover. Parents may prioritize their children’s insurance, as reported by one insured participant whose husband is also uninsured:

Interviewer: *Why do you have health insurance for your children but not for yourself?*

Respondent: *The children are getting sick just like that. They are small, and they are sick. That is why [my husband and I] do it for them* (Frafra woman aged 30, mother of three).

## Discussion

*Kayayei* suffer from greater illness or injury than the general female population in Accra. Both the sample proportion (38.4%) and population estimates (23.2%) generated by this study exceed the 10.0% prevalence of illness/injury reported among females in Accra the 2 weeks preceding the GLSS6 (Ghana Statistical Service, 2014a). Although recently ill/injured participants desired health care, less than half reported seeking care. Participants were more likely to seek medical care for illnesses/injuries that affected their ability to carry a load, such as arm or leg pain, than for illnesses like fever. Accessing formal health services in Accra requires money, time away from work, and for many migrants from northern Ghana, a translator who speaks Twi and/or English. An inability to offer patients the services of a translator in multilingual countries like Ghana can effectively exclude from care those internal migrants who do not speak the dominant language(s) at their destination.

A faster, easier and less expensive alternative is to seek care and medication directly from informal providers. Women are aware that such an approach involves a certain degree of risk, but a lack of financial resources restricts choices of safer options. The safety and efficacy of unlicensed medication sold by a peddler is unknown, and chemical shop employees may not be appropriately trained to prescribe drugs.

Financial barriers overwhelmingly limit *kayayei* migrants from seeking health care, influencing migrants’ decisions to seek no care or influencing from whom/where migrants seek care. Estimates suggest that approximately half of *kayayei* migrants in Accra are currently insured; yet, only 10.9% are estimated to have a health insurance card with them. Nearly four times as many members of Ghana’s population (39.0%) are valid card holding members in the NHIS (National Development Planning Commission, 2015). A lack of money prevents *kayayei* migrants from registering with the NHIS or renewing expired health insurance policies. This inability to access health insurance is compounded by the fact that *kayayei* migrants find it difficult to receive insurance exemptions.

Insured *kayayei* migrants may struggle to utilize their health insurance when cards are forgotten in the north or lost to fire and theft in Accra. For migrants who forgot or lost their insurance cards and those who cannot afford replacement cards, biometric and fingerprint data collected by Ghana’s National Health Insurance Agency (NHIA) may have the potential to confirm insurance status and permit access to health care at health facilities; such data are already used by the NHIA to improve identity checks. Fingerprint identification systems have also been implemented in Ghana as a method for linking community data from the Kintampo Health and Demographic Surveillance System to hospital data (Odei-Lartey *et al.*, 2016) and as a method for verifying voters by the Electoral Commission (Yakubu and Adjei, 2014); however, these experiences suggest that fingerprint identification works best when combined with other identification methods.

A lack of money also restricts both insured and uninsured migrants from seeking formal health services due to the unpredictable nature of out-of-pocket expenses. Out-of-pocket health expenditures push ∼380 000 Ghanaians into poverty each year (Garshong and Akazili, 2015), including participants in this study for whom these expenses served as a trigger to migrate. Ghana’s NHIS has the potential to mitigate such catastrophic health expenses, but information about the health insurance scheme needs to be better communicated. Some migrants were unaware that health insurance purchased in the north would work in the south. Others did not know that lost or forgotten health insurance cards could be replaced for a fee without purchasing a new policy. Pregnant women and children are perceived to benefit more from health insurance exemptions than the poor, in part due to an unclear definition of ‘indigents’ in the NHIS policy (Agyepong *et al.*, 2016). The NHIS indigent exemption is implemented inadequately and excludes many impoverished citizens. With *kayayei* migrants experiencing homeless in Accra and underemployed or unemployed on slow market days, the indigent exemption in Accra needs to be revisited.

This research updates the discussion on health status and health insurance among migrants employed in Ghana’s informal sector and may be generalizable to other migrant groups working in Ghana’s informal sector. Women employed in cross-border trading, casual agricultural labourers migrating south from northern Ghana and Burkina Faso, and female and child miners employed in artisanal and small-scale gold mining may experience similar barriers to health care and health insurance. These hazardous occupations may also place these populations at greater risk of illness or injury than the general population. Like *kayayei*, women employed in cross-border trading are concerned with survival needs; these traders may self-medicate using over-the-counter medications due to difficulty taking time away from work and limited access to nearby health facilities (Wrigley-Asante, 2013).

The findings from this study complement findings from elsewhere. A cross-sectional study among migrant workers in China found that migrants’ inability to pay for health care resulted in lower levels of inpatient care utilization (Mou *et al.*, 2009). Much like *kayayei* migrants, the China study’s young, low-paid, less educated female migrants were more likely to be uninsured and to pay for health care out of pocket (Mou *et al.*, 2009). In Vietnam, a cross-sectional study among migrants and non-migrants found lower levels of health care utilization among migrants than non-migrants with the lowest levels of utilization reported among seasonal migrants (Le *et al.*, 2015). Seasonal migration is common among *kayayei* migrants who may migrate on school holidays or between harvests.

In Indonesia, where national health insurance expansion efforts have struggled to insure informal sector workers through contributory or subsidized schemes, recent research stresses the importance of understanding how socio-demographic factors, health behaviours and health status affect health insurance coverage (Idris *et al.*, 2017). These factors affect health insurance coverage among *kayayei* migrants in Ghana and even workers in high-income countries like the Czech Republic, where linguistic barriers and a lack of awareness about public health insurance have excluded eligible immigrants from the public health insurance system (Dzúrová *et al.*, 2014). Equal access to health care requires better understanding determinants of health insurance coverage among migrant populations, raising greater awareness of health insurance and its costs, and in the case of Ghana, clarification that health insurance cards work nationwide. Similarities in health-related disadvantages arising from informal sector employment, migrant status and gender reported by studies in Ghana and elsewhere suggest that the findings among *kayayei* migrants are likely generalizable to other migrant groups within Ghana and within other LMIC settings.

Health insurance is crucial for accessing care, but migrant-friendly services also help improve service uptake. In Thailand, the public health ministry developed a model for insured and uninsured migrants that uses volunteer community health workers, mobile clinics for migrant communities, bilingual signs and information and workplace outreach services (Tangcharoensathien *et al.*, 2017). This model has promoted health awareness among migrants and improved service uptake by helping migrants navigate health services (Tangcharoensathien *et al.*, 2017).

### Limitations

For RDS analyses, the sample distribution within most variables had stabilized or reached equilibrium, indicating that the selection of ‘seeds’ or participants from whom the sample originated did not bias the final sample with regards to these variables (Heckathorn, 1997, 2002); however, ethnicity and religion were two notable exceptions. Achieving equilibrium for ethnic group would have required running the survey for an average of 54 ‘waves’ (rounds of people recruited from the initial seed), exceeding the resources of this study. Religion, a variable closely linked to ethnic group in this setting, would have similarly required extensive waves. The final sample of *kayayei* may be biased with regards to religion and ethnic group. Census data, however, suggests that the potential for sampling bias might not be that significant. Ethnic and religious distributions among northern female migrants in Accra from the census data are comparable to the population in this study.

## Conclusion

It is paradoxical that migrants from northern Ghana, where the concentration of health facilities is lowest, cannot and do not access formal health services in Accra, where Ghana’s concentration of health facilities is highest. Financial barriers to health care services continue to burden *kayayei* migrants in Accra, even among migrants who report being insured under Ghana’s NHIS or who qualify for insurance exemptions. Linguistic differences between providers and patients further restrict migrants’ access to health care. These barriers led participants to seek no care or to seek care from informal providers.

In order to better meet migrants’ health care needs, the findings from this study support the following policy recommendations:

- Policymakers should revisit implementation of the NHIS’s indigent exemption and should consider broadening the eligibility criteria. Developing an official mechanism for community organizations like the KYA to provide District Mutual Health Insurance Scheme Managers with lists of poor individuals who most need exemptions could help ensure that the indigent exemption is effectively applied to those most in need.
- To improve equity, policymakers should consider mandating that the poorest districts enrol higher percentages of individuals receiving ‘core poor’ or indigent exemptions than the national average. Such an approach would likely benefit many participants in this study, particularly those who cited family medical expenses as a driver of their migration.
- The formal health system must better incorporate culturally appropriate care into the provision of health services. For example, targeted training of health care professionals at medical facilities near informal settlements/slums could help reduce xenophobia and biases, such as providers assuming that migrant patients were beaten in response to theft. Providing trained interpreters would also facilitate improved communication and understanding between providers and migrant patients.

It is time to better design health systems to help migrants, poor people and the informal sector access health care. Achieving UHC requires increased focus on equity as well as demographic adaptations to ensure access to affordable, quality health services across domestic borders. Migrants are uniquely positioned to test the strengths and weaknesses of UHC across internal and international borders. Their efforts to access care in multiple districts or regions can illustrate points of weakness within the system but also points of intervention for stronger health systems. With internal migration on the rise in many settings, health systems must recognize the varied needs of populations in multi-ethnic and multilingual countries if countries are to achieve health coverage that is truly universal.
